# The Pathological Changes Which May Occur in Uterine Fibroids

**Published:** 1909-08-14

**Authors:** 


### Gynæcology.

### THE PATHOLOGICAL CHANGES WHICH MAY OCCUR IN UTERINE FIBROIDS—(*continued*).

Infection of a uterine fibroid is always a serious matter, and under some circumstances may be a direct menace to life. It is fortunate that interstitial fibroids are not easily infected, but even this occurs sometimes. Commonly, however, it is a submucous fibroid being extruded by uterine contractions through the os uteri, or a pedunculated fibroid projecting into the vagina, which becomes infected. Infection is more easily brought about when the fibroid projects into the vagina for several reasons. First, the bacteria are at hand there and are helped to grow by the presence of unusual secretions set up by the irritation of the fibroid; secondly, the pedunculated fibroid is subject to injury in the vagina; and, thirdly, it is liable to become constricted at its base and partially strangulated. When a fibroid thus becomes infected it is quite common for a patch of gangrene to occur on the surface, surrounded by a hyperæmic zone, and giving rise to a most offensive discharge. The gangrenous process need not even be limited, but may spread to a large area of the tumour with gradual disintegration and the formation of a ragged sloughing cavity. This condition is always accompanied by pyrexia and some general toxæmia, the degree depending on the severity of the gangrenous process.

Interstitial fibroids become infected in two ways commonly; either from the uterine cavity after a labour in which the uterus has been infected, or from the intestines when adhesions have occurred between the latter and the uterus. In either of these cases the infective process is liable to give rise to pus formation in and around the tumour and local peritoneal infection with abscesses may occur as well. In such conditions the general symptoms are very severe, resembling a true septicæmia, but as a rule not ending fatally if drainage can be established and the infected tumour removed. There are several cases on record in which the tumour has been extruded into the uterine cavity, become quite detached by the suppurative process, and expelled into the vagina.

The general treatment of infected fibroids must be complete removal, and as a rule when the tumour hangs into the vagina this is not a very serious matter. When, however, the tumour is only beginning to be extruded from the uterus and has to be enucleated through the dilated cervix, precautions must be taken so as not to infect the bed from which it is removed. It is best to cut away the gangrenous portions first with scissors and forceps, and then to disinfect the raw surface with the actual cautery and the surrounding parts with ether soap, followed by a strong lysol solution thoroughly scrubbed over the parts with sterile absorbent wool. After enucleation of such tumours the uterine cavity should be well washed out and finally plugged with 10 per cent. iodoform gauze which has been soaked in an antiseptic. If the projecting tumour is only one of several, a hysterectomy becomes necessary, and great care must be taken to prevent infection of the peritoneum during the operation. The best plan will be to cut away the gangrenous area per vaginam, and then disinfect the vagina and cervix with ether soap and lysol prior to the abdominal hysterectomy. In such cases it is better to perform pan-hysterectomy and drain the peritoneum per vaginam, rather than leave the cervix behind, which will be difficult to disinfect or drain completely.

Calcification of uterine fibroids is liable to occur in comparatively small tumours, generally in elderly women past the menopause. They are often found in the post-mortem room, having caused no symptoms or having been forgotten. However, they sometimes give rise to great pain, partly on account of their weight, of pressure on surrounding organs, or of their unyielding consistence. In these circumstances they sometimes call for removal; the operation of hysterectomy under such circumstances provides no particular difficulties. There is no reason known for the calcification of these tumours, but it may be remembered that calcareous changes are liable to occur in all tumours which are largely fibrous in nature. For instance, it is not uncommonly seen in fibroma of the ovary, and there is some reason to believe that the change is associated with deficient blood supply and possibly with partial torsion of the pedicle.

Sarcomatous degeneration is the most debated degeneration which has been described in connection with fibroids, and there are very few cases of the kind which cannot be viewed with suspicion. Strictly, the term means the transformation of the muscle cells or fibrous tissue in a fibroid into sarcomatous elements. This actual change can very seldom be demonstrated, but it has been claimed to have been seen by some observers, and on the whole we have not enough evidence to say that it does not occur. The more common condition demonstrable, however, is a large tumour, obviously malignant, because it has invaded organs outside the uterus in which masses of tissue occur which are clearly fibromyomatous and other masses which are as clearly sarcomatous. As a rule, however, it is not possible to trace the transition of the one to the other. It must not be forgotten that a sarcoma may arise independently in a uterus which is already the seat of fibroids, either in the muscular wall of the uterus or in the endometrium. From either of these situations the malignant growth may attack and more or less destroy a fibroid without in any way being a degeneration of it. It is possible, too, that a sarcoma may actually arise in a fibroid without being a degeneration of it, but this is much more difficult of proof. Finally it cannot be denied that some sarcomata are of very slow growth at first and may be mistaken for fibroids. Sarcoma in association with fibroids can only be surmised on account of rapid growth, grave constant hæmorrhage, pain and emaciation. Under these circumstances a radical operation is necessary, and the whole uterus and cervix should be removed.

